# Genome Sequence of *Tumebacillus flagellatus* GST4, the First Genome Sequence of a Species in the Genus *Tumebacillus*

**DOI:** 10.1128/genomeA.01189-14

**Published:** 2014-11-13

**Authors:** Qing-Yan Wang, Neng-Zhong Xie, Yan-Yan Huang, Li-Fu Song, Qi-Shi Du, Bo Yu, Dong Chen, Ri-Bo Huang

**Affiliations:** a National Engineering Research Center for Non-Food Biorefinery, State Key Laboratory of Non-Food Biomass Energy and Enzyme Technology, Guangxi Academy of Sciences, Nanning, China; b CAS Key Laboratory of Microbial Physiological and Metabolic Engineering, Institute of Microbiology, Chinese Academy of Sciences, Beijing, China; c Institute of Bioprocess and Biosystems Engineering, Hamburg University of Technology, Hamburg, Germany

## Abstract

We present here the first genome sequence of a species in the genus *Tumebacillus*. The draft genome sequence of *Tumebacillus flagellatus* GST4 provides a genetic basis for future studies addressing the origins, evolution, and ecological role of *Tumebacillus* organisms, as well as a source of acid-resistant amylase-encoding genes for further studies.

## GENOME ANNOUNCEMENT

With an increase in the demand for enzymes in recent decades, the global market for industrial enzymes has grown significantly and was estimated to be worth $7 billion in 2013 (1). Amylases are one of the most important kinds of enzymes and play an essential role in many fields, such as in the food, chemical, pharmaceutical, textile, feed, and detergent industries. Commercially, this class of industrial enzymes constitutes approximately 25 to 33% of the global enzyme market (2–5).

As the pH of native starch solution is acidic (pH 3.2 to 4.5), using neutral or alkaline amylases for starch hydrolysis results in significant process operating costs for pH adjustments on a large scale. Therefore, acid-resistant amylases are the preferred enzymes for starch hydrolysis in several industrial processes (6, 7). For example, ethanol industries mainly use commercially available acid-resistant glucoamylase, α-amylase, and pullulanase (8). Owing to their industrial importance, there is an ongoing interest in the isolation of new acid-resistant amylases with industrial potential (5).

Organisms belonging to the genus *Tumebacillus* are Gram-positive, aerobic, rod-shaped, spore-forming bacteria (9–11). To date, none of genome sequences of *Tumebacillus* strains has been reported. In our investigation of bacteria that produce acid-resistant starch-hydrolyzing enzymes, we isolated an acid-tolerant *Tumebacillus flagellatus* strain GST4 (DSM 25748) from the wastewater of a cassava starch factory, and this organism was found to represent a novel species of *Tumebacillus* (11). Because of the complex structure of starch, starch-degrading bacteria require a combination of various starch-hydrolyzing enzymes for depolymerizing starch to smaller sugars and monosaccharides (2, 12). We therefore sequenced and analyzed the genome of strain GST4 to provide the genetic basis for further study.

Here, we report the draft genome sequence of *T. flagellatus* GST4, which was obtained using the Illumina HiSeq 2500 next-generation DNA platform. A total of 3,814,788 reads were generated, reaching a depth of 157-fold genome coverage. The reads were assembled into 108 contigs (*N*_50_ contig size, 125,340 bp) using the program Velvet (13). The assembled data were deposited in the NCBI nucleotide sequence database and annotated with the NCBI Prokaryotic Genomes Automatic Annotation Pipeline (PGAAP). The draft genome of *T. flagellatus* GST4 includes 4,874,793 bp containing 4,577 open reading frames (ORFs), 9 rRNA genes, and 33 tRNA genes, with a G+C content of 56.5%. The annotation results showed that 3,208 proteins have positive biology functions, 1,915 proteins have KEGG orthologs, and 3,227 proteins have Clusters of Orthologous Groups (COG) classifications.

The availability of the first genome sequence of an organism belonging to the genus *Tumebacillus* helps to clarify the evolutionary status and ecological role of the genus and provides a genetic basis for analyses of the acid tolerance and starch-hydrolyzing activities of *T. flagellatus* GST4. Additionally, genes encoding various starch-hydrolyzing enzymes, such as α-amylase, isoamylase, glucoamylase, pullulanase, neopullulanase, and amylopullulanase, were successfully annotated, providing a genetic basis for exploiting new acid-resistant amylases suitable for industrial processes.

### Nucleotide sequence accession numbers.

This whole-genome shotgun project has been deposited in DDBJ/ENA/GenBank under the accession no. JMIR00000000. The version described in this paper is the first version, JMIR01000000.
